# Transarterial chemoembolization combined with molecularly targeted agents plus immune checkpoint inhibitors for unresectable hepatocellular carcinoma: a retrospective cohort study

**DOI:** 10.3389/fimmu.2023.1205636

**Published:** 2023-07-31

**Authors:** Nan Jiang, Binyan Zhong, Jintao Huang, Wanci Li, Shuai Zhang, Xiaoli Zhu, Caifang Ni, Jian Shen

**Affiliations:** Department of Interventional Radiology, The First Affiliated Hospital of Soochow University, Suzhou, Jiangsu, China

**Keywords:** transarterial chemoembolization, targeted therapy, immune therapy, unresectable hepatocellular carcinoma, combination therapy

## Abstract

**Purpose:**

To retrospectively evaluate and compare treatment effectiveness and safety between transarterial chemoembolization (TACE) combined with molecularly targeted agents plus immune checkpoint inhibitors (TACE+T+I) and TACE combined with molecularly targeted agents (TACE+T) for unresectable hepatocellular carcinoma (uHCC).

**Methods:**

We retrospectively analyzed the data of patients with unresectable HCC from January 2018 to June 2022. The patients were screened based on the inclusion criteria and were divided into the triple combination group (TACE+T+I) and the double combination group (TACE+T). The primary outcomes were overall survival (OS), progression-free survival (PFS), and adverse events (AEs). The secondary outcomes were objective response rate (ORR) and disease control rate (DCR). Risk factors associated with PFS and OS were determined by Cox regression analysis.

**Results:**

A total of 87 patients were enrolled in this study, including 42 patients in the TACE+T+I group and 45 patients in the TACE+T group. Over a median follow-up of 29.00 and 26.70 months, patients who received TACE+T+I therapy achieved a significantly longer median OS (24.00 *vs.* 21.40 months, *p* = 0.007) and median PFS (9.70 *vs.* 7.00 months, *p* = 0.017); no grade 4 AEs or treatment-related death occurred in the two groups. Grade 3 AEs attributed to systemic agents in the two groups showed no significant difference (19.0% *vs.* 15.6%, *p* = 0.667). Patients in the TACE+T+I group demonstrated better tumor response when compared with patients in the TACE+T group, with an ORR of 52.4% *vs.* 17.8% (*p* = 0.001). No significant difference was observed in DCR between the two groups (83.3% *vs.* 77.8%, *p* = 0.514*)*. Cox regression analysis showed that only the treatment method was an independent factor of OS, and both age and treatment method were independent factors related to PFS.

**Conclusion:**

Compared with TACE plus molecularly targeted agents (TACE+T), the triple therapy (TACE+T+I) could improve survival and tumor response in unresectable HCC with manageable toxicities.

## 1 Introduction

Hepatocellular carcinoma (HCC) accounts for 75%–85% of primary liver cancer (PLC) (1). It is the third leading cause of cancer-related death. However, in China, more than 80% of HCC patients are diagnosed in intermediate and advanced stages at the first time of diagnosis, missing the opportunity to receive radical treatments (2). For these patients, treatment recommendations include transarterial chemoembolization (TACE) and systemic therapies according to the Barcelona Clinic Liver Cancer (BCLC) staging system (3).

TACE has been a key component of local treatments for patients with HCC, which is recommended for the intermediate stage of unresectable HCC (uHCC) (3). Due to the limitations of TACE such as activating hypoxia-inducible factor-1α (HIF-1α) (4), TACE combined with other local treatments or systemic treatments is also recommended (3, 5). In the treatment of advanced HCC, the guidelines previously recommended sorafenib and lenvatinib as first-line treatment. Recently, atezolizumab plus bevacizumab (T+A) and tremelimumab plus durvalumab have also been included as first-line treatments for the stage of BCLC-C (6, 7).

With the success of immune checkpoint inhibitors (ICIs) in the treatment of many malignancies, the era of immunotherapy for HCC has begun. However, in reality, most patients receiving ICIs monotherapy have not achieved significant clinical response (8). Recently, some randomized controlled trials (RCTs) analyzing the combination of molecularly targeted agents and ICIs had positive results (6, 9, 10), indicating that other therapies are needed to be combined with ICIs to attain a breakthrough in dealing with HCC. A systemic review showed that triple therapy of TACE, tyrosine kinase inhibitors (TKIs), and ICIs would provide a clinical benefit for uHCC in both short- and long-term outcomes without increasing severe AEs (11). Therefore, the triple therapy of TACE combined with molecularly targeted agents and ICIs in the treatment of HCC has a synergistic effect and may achieve better efficacy. Several RCTs concerning the triple combination treatment are currently underway, and the results have not come through yet, but this therapy has already been gradually conducted in real-world practice. Our study retrospectively evaluated the effectiveness and safety of TACE combined with targeted and immune agents for intermediate and advanced HCC by comparing with TACE combined with molecularly targeted agents in order to provide more options for HCC treatments.

## 2 Material and methods

### 2.1 Study design and patients

Patients who were diagnosed with HCC and classified as intermediate and advanced stage according to the China liver cancer staging (CNLC) and BCLC and treated with TACE from January 2018 to June 2022 were enrolled. The inclusion criteria were 1) aged from 18 to 75; 2) BCLC stage B or C; 3) patients received TACE plus molecularly targeted therapy with/without ICIs, with an interval between TACE and the first use of molecularly targeted therapy with/without ICIs of ≤1 month; 4) the systemic agents included first-line and second-line recommended; 5) computed tomography (CT) or magnetic resonance (MR) images have at least one measurable lesion as defined by modified Response Evaluation Criteria in Solid Tumors (mRECIST); 6) Eastern Cooperative Oncology Group (ECOG) Performance Status (PS) of 0 or 1. The exclusion criteria were 1) Child-Pugh class C, 2) had received other local treatments before TACE, 3) had a contraindication to TACE or molecularly targeted therapy/ICIs, 4) predicted life expectancy of <3 months, and 5) incomplete clinical data.

### 2.2 Treatment

TACE was performed by interventional radiologists with more than 10 years of experience in the procedure. Pirarubicin 20 mg combined with oxaliplatin 0.1 g was used for conventional transarterial chemoembolization (cTACE) (the use of pirarubicin was determined by patients’ electrocardiogram results). Epirubicin 50 mg was used for drug-eluting bead transarterial chemoembolization (DEB-TACE). The need for repeated TACE was evaluated according to the results of enhanced CT or MR and tumor indicators with “on demand” mode.

All systemic treatments were started at standard doses in light of the instructions for use. The molecularly targeted agents included sorafenib 0.4 g orally twice daily, lenvatinib 12 mg (≥60 kg) or 8 mg (<60 kg) orally once daily, apatinib 750 mg orally once daily, regorafenib 160 mg orally once daily (for the first 21 days of each 28-day cycle), and bevacizumab 15 mg/kg intravenously every 3 weeks. All ICIs were applied intravenously every 3 weeks including camrelizumab, sintilimab, pembrolizumab, tislelizumab, and atezolizumab with a dosage of 200 mg (except for 1,200 mg for atezolizumab). Dose reduction or treatment termination would be determined according to the adverse drug reactions of patients. Patients received systemic treatments until disease progression or intolerable toxic reactions. Afterward, subsequent treatments, mainly including radiotherapy, ablation, hepatic arterial infusion chemotherapy (HAIC), and palliative care according to the individual inclination of patients, were introduced.

The methods of TACE or the types of systemic agents were selected according to the tumor conditions and willingness of patients. Specifically, physicians illustrated clearly the patients’ condition of illness and the alternatives of treatment and medicine, and then the patients’ families made the decisions depending on the physicians’ suggestions, financial burden, and other considerations. In some of the patients, a multidisciplinary team is involved in the decision-making.

### 2.3 Follow-up and evaluation

Enhanced CT/MR imaging and laboratory tests were conducted every 2 months after TACE. The tumor response of all patients was evaluated according to the mRECIST and classified as complete response (CR), partial response (PR), stable disease (SD), and progressive disease (PD). All patients were followed up until 32 December 2022 or until death. The primary outcomes of this study were overall survival (OS), progression-free survival (PFS), and adverse events (AEs). OS was defined as the time from initial treatment to death or the end of follow-up. PFS was defined as the time from the initial treatment to the first occurrence of tumor progression or the end of follow-up or death. The initial treatment was defined as the first combination of TACE with systemic therapy. TACE conducted more than 1 month before baseline was considered as the previous history. Secondary outcomes included objective response rate (ORR), which is defined as the proportion of patients who achieved CR or PR, and disease control rate (DCR), which is defined as the proportion of patients who achieved CR, PR, or SD.

AEs were assessed and recorded in accordance with the Common Terminology Criteria for Adverse Events (CTCAE) Version 5.0.

### 2.4 Statistical analysis

Statistical analysis was performed using SPSS software (version 27.0). Student’s t-test or the Mann–Whitney U test was used to analyze continuous data. Chi-square or Fisher’s exact test was used to assess categorical parameters, which were presented as frequency and percentages. The Kaplan–Meier curve was used to calculate the median OS and median PFS, and comparison was performed by log-rank test. Independent factors were identified by Cox proportional hazards regression analysis. *p*-Values less than 0.05 were considered statistically significant.

## 3 Results

### 3.1 Patient clinical characteristics

**Figure 1:**
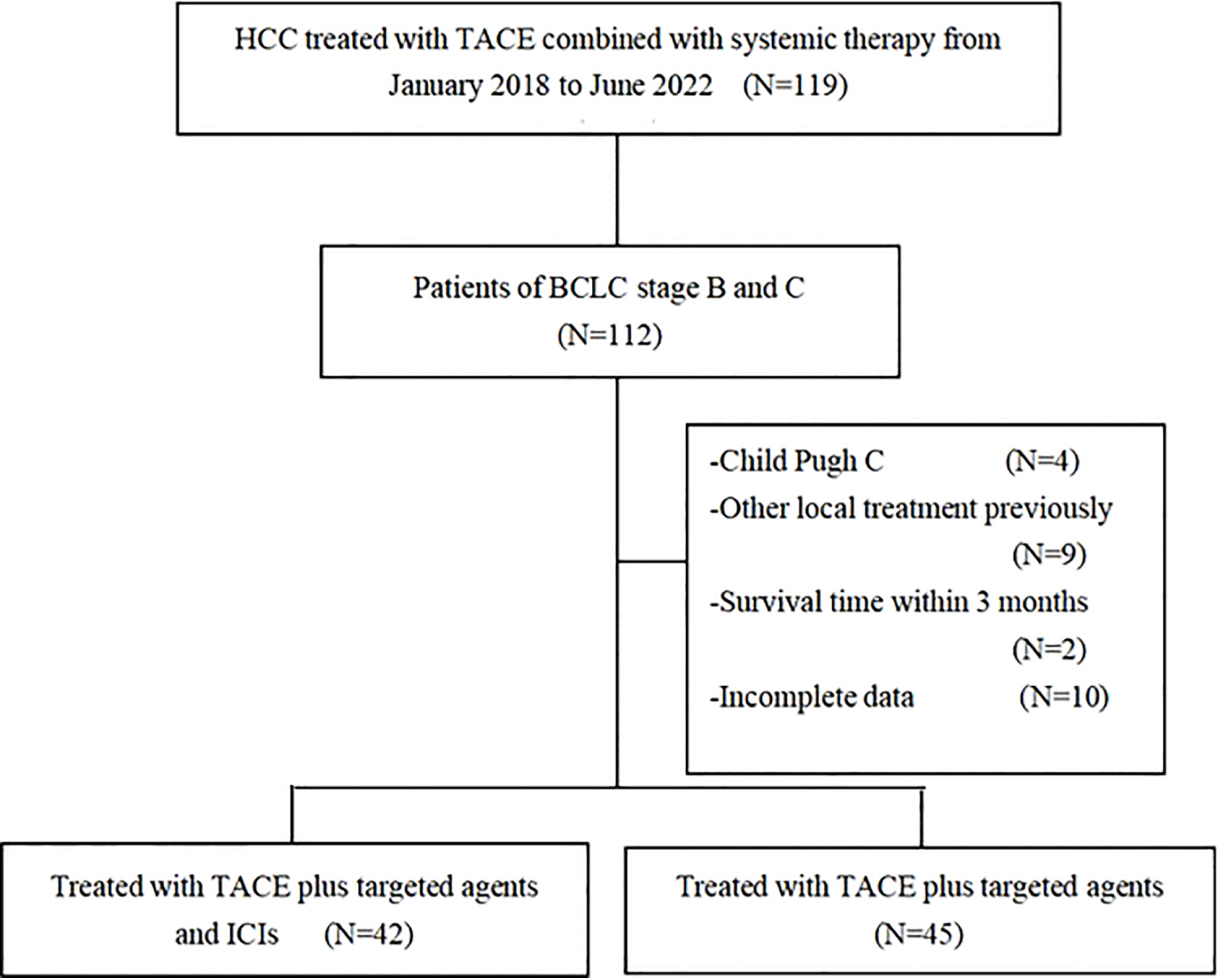
Flow chart of patients.

After screening (**Figure 1**), 87 eligible patients with intermediate and advanced HCC were enrolled in this study, including 42 who received TACE+T+I and 45 who received TACE+T. Patients’ baseline characteristics are summarized in **Table 1**. The age, gender, hepatitis B virus (HBV) infection, previous surgery, previous TACE, tumor distribution, tumor size, vascular invasion, extrahepatic metastasis, Child-Pugh grade, albumin-bilirubin (ALBI) score, ECOG PS score, alpha-fetoprotein (AFP), and Protein Induced by Vitamin K Absence or Antagonist II (PIVKA-II) were analyzed and showed no significant differences between the two groups (*p* > 0.05).

**Table 1:** Baseline characteristics of patients.

	TACE+T+I (n = 42)	TACE+T (n = 45)	*p*-Value
Age	61.71 ± 9.48	61.24 ± 12.10	0.841
Gender			0.101
Male	33 (78.57%)	41 (91.11%)	
Female	9 (21.43%)	4 (8.89%)	
HBV			0.888
(+)	35 (83.33%)	38 (84.44%)	
(−)	7 (16.67%)	7 (15.56%)	
Previous surgery			0.363
Yes	12 (28.57%)	17 (37.78%)	
No	30 (71.43%)	28 (62.22%)	
Previous TACE			0.085
Yes	8 (19.05%)	16 (35.56%)	
No	34 (80.95%)	29 (64.44%)	
Tumor distribute			0.168
≤3	23 (53.49%)	18 (40.00%)	
>3	19 (45.24%)	27 (60.00%)	
Tumor size			0.519
≤5 cm	14 (33.33%)	18 (40.00%)	
>5 cm	28 (66.67%)	27 (60.00%)	
Largest tumor size	7.63 ± 6.57	6.82 ± 4.22	0.497
Vascular invasion			0.842
Yes	15 (35.71%)	17 (37.78%)	
No	27 (64.29%)	28 (62.22%)	
Extrahepatic metastasis			0.170
Yes	8 (19.05%)	4 (8.89%)	
No	34 (80.95%)	41 (91.11%)	
Child-Pugh grade			0.888
A (5–6)	35 (83.33%)	38 (84.44%)	
B (7–9)	7 (16.67%)	7 (15.56%)	
ALBI score			0.514
≤−2.60	7 (16.67%)	10 (22.22%)	
−2.60 to −1.39	35 (83.33%)	35 (77.78%)	
ECOG PS			0.561
1	17 (40.48%)	21 (46.67%)	
0	25 (59.52%)	24 (53.33%)	
AFP			0.467
≤400 μg/ml	31 (73.81%)	30 (66.67%)	
>400 μg/ml	11 (26.19%)	15 (33.33%)	
PIVKA-II			0.573
≤900 mAU/ml	18 (42.86%)	22 (48.89%)	
>900 mAU/ml	24 (57.14%)	23 (51.11%)	

HBV, hepatitis B virus; TACE, transarterial arterial chemoembolization; ALBI, albumin-bilirubin; ECOG PS, Eastern Cooperative Oncology Group Performance Status; AFP, alpha-fetoprotein; PIVKA, Protein Induced by Vitamin K Absence or Antagonist II; (+), positive; (-), negative.

### 3.2 Agents’ duration and TACE exposure

Patients in the TACE+T+I and TACE+T groups underwent 2.50 ± 1.57 (range 1–8) and 2.58 ± 1.23 (range 1–7) TACE sessions, respectively (*p* = 0.797). In the TACE+T+I group, molecularly targeted agents and ICIs were administered for an average of 15.58 ± 9.58 months and 9.43 ± 7.85 cycles, respectively. In the TACE+T group, the average length of molecularly targeted agents administration was 12.73 ± 7.90 months (*p* = 0.132).

### 3.3 Survival

**Figure 2:**
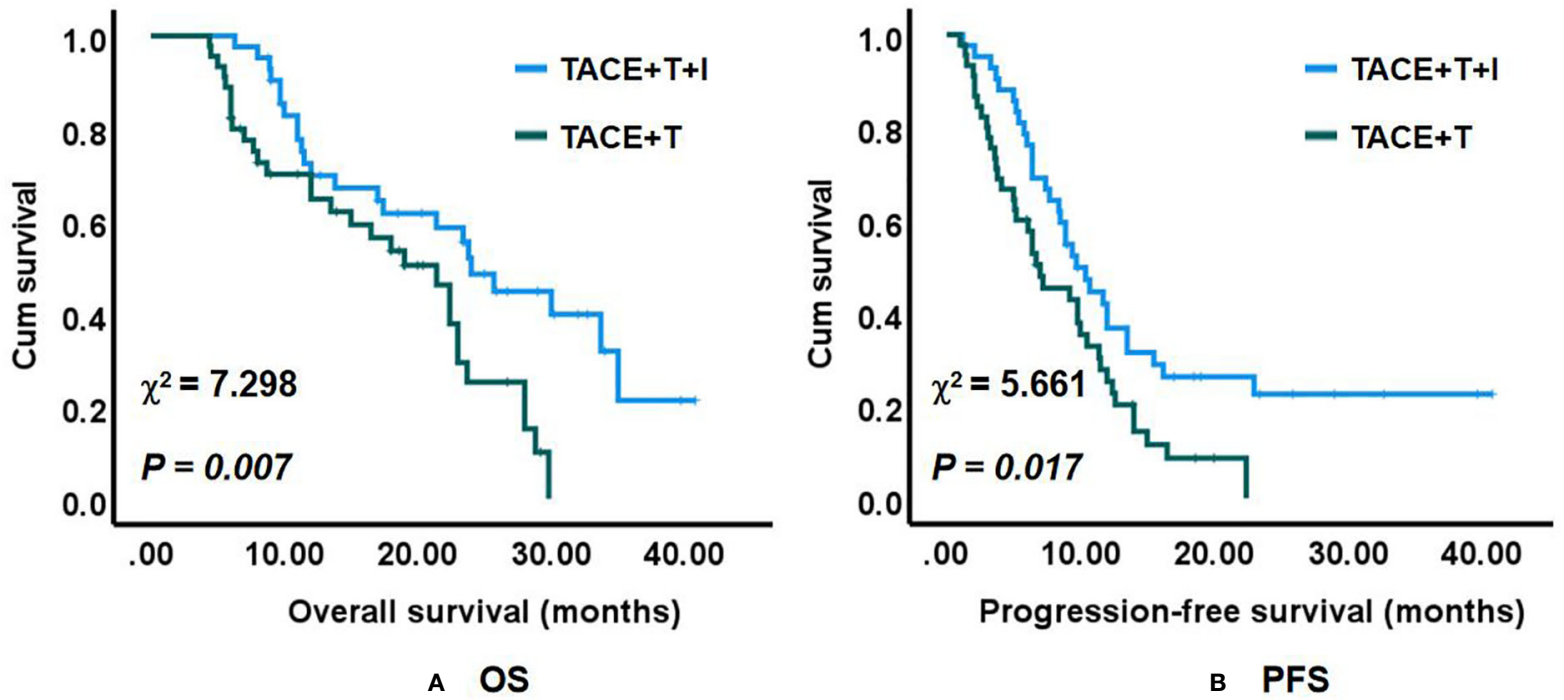
**(A)** OS and **(B)** PFS.

At the data cutoff, the median follow-up time was 29.00 months for the TACE+T+I group and 26.70 months for the TACE+T group. A total of 19 (45.2%) patients in the TACE+T+I group and 15 (33.3%) patients in the TACE+T group survived during the follow-up. Compared with the TACE+T group, median OS (**Figure 2A**) was significantly prolonged in the TACE+T+I group [24.00 months (95% CI: 20.41–27.59) *vs.* 21.40 months (95% CI: 16.15–26.66), *p* = 0.007] according to log-rank analysis. Median PFS (**Figure 2B**) was 9.70 months (95% CI: 7.47–11.93) for the TACE+T+I group and 7.00 months (95% CI: 3.88–10.12) for the TACE+T group, also with significant statistical difference (*p* = 0.017).

In patients with BCLC stage B (N = 22 *vs.* 25), the median OS was 33.70 months (95% CI: 17.87–49.53) in the TACE+T+I group and 22.40 months (95% CI: 20.75–24.05) in the TACE+T group, and the difference has statistical significance (*p* = 0.040). The median PFS in these two groups were comparable [10.4 months (95% CI: 5.89–14.92) *vs.* 9.20 months (95% CI: 6.08–12.33), *p* = 0.117]. In patients with BCLC stage C (N = 20 *vs.* 20), the median OS and PFS were 17.40 months (95% CI: 2.47–32.34) and 8.9 months (95% CI: 6.41–11.39), respectively, in the TACE+T+I group and 8.70 months (95% CI: 3.08–14.32) and 5.1 months (95% CI: 4.66–5.54), respectively, in the TACE+T group; the OS and PFS between the two groups were significantly different (*p* = 0.023, *p* = 0.038).

### 3.4 Tumor response for treatment

The results of the best tumor response in the two groups are displayed in **Table 2**. In accordance with the mRECIST standard; 4/18/13 patients in the TACE+T+I group had the best tumor response of CR/PR/SD compared with 0/8/27 for the TACE+T group. The ORR in the TACE+T+I group was significantly higher than that in the TACE+T group [52.4% (95% CI: 37.3%–67.5%) *vs.* 17.8% (95% CI: 6.6%–28.9%), *p* = 0.001]. The DCR rate in the two groups demonstrated no statistical difference [83.3% (95% CI: 72.1%–94.6%) *vs.* 77.8% (95% CI: 65.6%–89.9%), *p* = 0.514].

**Table 2:** Best overall response according to mRECIST.

	TACE+T+I (N = 42)	TACE+T (N = 45)	*p*-Value
Best response			
CR	4 (9.5%)	0 (0%)	
PR	18 (42.9%)	8 (17.8%)	
SD	13 (31.0%)	27 (60.0%)	
ORR	52.4% (95% CI: 37.3%–67.5%)	17.8% (95% CI: 6.6%–28.9%)	**0.001**
DCR	83.3% (95% CI: 72.1%–94.6%)	77.8% (95% CI: 65.6%–89.9%)	0.514

mRECIST, modified Response Evaluation Criteria in Solid Tumors; CR, complete response; PR, partial response; SD, stable disease; ORR, objective response rate; DCR, disease control rate. P-Values less than 0.05 are shown in bold.

### 3.5 Independent factors affecting OS and PFS

Univariate and multivariate Cox proportional hazards regression analyses were performed to evaluate the independent predictors of OS. *p* > 0.1 was considered not independently associated with OS/PFS in univariate analysis. The results in **Table 3** indicated that only the different treatment method was the independent factor of OS (*p* = 0.004, HR = 2.377, 95% CI: 1.317–4.290), and the treatment of TACE+T+I was a strongly protective factor. After analysis, age and treatment method were the independent factors of PFS (*p* = 0.037, HR = 0.594, 95% CI: 0.364–0.970, *p* = 0.020, HR = 1.788, 95% CI: 1.097–2.915). Age < 65 predicted a shorter time before progression, and the treatment of TACE+T+I also was a protective factor of PFS (**Table 4**).

**Table 3:** Univariate and multivariate analyses of risk factors for overall survival.

Characteristics	Univariate analysis			Multivariate analysis		
	HR	95% CI	*p*-Value	HR	95% CI	*p*-Value
Gender (male *vs.* female)	0.890	0.400–1.979	0.775			
Age (years) (≥65 *vs.* <65)	0.642	0.2368–1.122	0.120			
HBV (yes *vs.* no)	1.035	0.464–2.310	0.933			
Previous surgery (yes *vs.* no)	0.656	0.369–1.166	0.151			
AFP (ng/ml) (>400 *vs.* ≤400)	1.015	0.555–1.858	0.961			
PIVKA (ng/ml) (>900 *vs.* ≤900)	1.244	0.720–2.150	0.434			
Child-Pugh grade (B *vs.* A)	1.789	0.892–3.591	0.102			
ALBI grade (2 *vs.* 1)	1.479	0.683–3.202	0.321			
ECOG PS score (1 *vs.* 0)	1.405	0.818–2.414	0.218			
BCLC stage (C *vs.* B)	1.972	1.134–3.429	0.016	2.270	0.905–5.693	0.081
Vascular invasion (yes *vs.* no)	1.887	1.075–3.314	0.027	0.860	0.334–2.218	0.756
Extrahepatic metastasis (yes *vs.* no)	1.605	0.803–3.207	0.181			
Previous TACE (yes *vs.* no)	0.918	0.513–1.640	0.772			
Tumor distribution (>3 *vs.* ≤3)	1.546	0.870–2.749	0.138			
Tumor size (cm) (>5 *vs.* ≤5)	1.645	0.925–2.925	0.090	1.763	0.980–3.169	0.058
**Treatment (TACE+T** *vs.* **TACE+T+I)**	2.164	1.216–3.852	0.009	2.377	1.317–4.290	**0.004**

HR, hazard ratio; CI, confidence interval; HBV, hepatitis B virus; AFP, alpha-fetoprotein; PIVKA, Protein Induced by Vitamin K Absence or Antagonist II; ALBI, albumin-bilirubin; ECOG PS, Eastern Cooperative Oncology Group Performance Status; TACE, transarterial chemoembolization; TACE+T+I, TACE + targeted agents + immune checkpoint inhibitors; TACE+T, TACE + targeted agents. P-Values less than 0.05 in multivariate analysis are shown in bold.

**Table 4:** Univariate and multivariate analyses of risk factors for progression-free survival.

Characteristics	Univariate analysis			Multivariate analysis		
	HR	95% CI	*p*-Value	HR	95% CI	*p*-Value
Gender (male *vs.* female)	1.257	0.639–2.472	0.508			
**Age (years) (≥65** *vs.* **<65)**	0.556	0.345–0.898	0.016	0.594	0.364–0.970	**0.037**
HBV (yes *vs.* no)	1.271	0.651–2.484	0.482			
Previous surgery (yes *vs.* no)	1.076	0.657–1.764	0.771			
AFP (ng/ml) (>400 *vs.* ≤400)	1.379	0.826–2.304	0.219			
PIVKA (ng/ml) (>900 *vs.* ≤900)	1.073	0.669–1.721	0.769			
Child-Pugh grade (B *vs.* A)	1.035	0.543–1.974	0.917			
ALBI grade (2 *vs.* 1)	0.991	0.531–1.849	0.977			
ECOG PS score (1 *vs.* 0)	1.265	0.788–2.031	0.331			
BCLC stage (C *vs.* B)	1.538	0.958–2.471	0.075	1.615	0.706–3.692	0.256
Extrahepatic metastasis (yes *vs.* no)	1.426	0.747–2.723	0.282			
Vascular invasion (yes *vs.* no)	1.520	0.937–2.464	0.090	0.914	0.390–2.141	0.836
Previous TACE (yes *vs.* no)	0.872	0.518–1.466	0.605			
Tumor distribution (>3 *vs.* ≤3)	1.150	0.717–1.844	0.562			
Tumor size (cm) (>5 *vs.* ≤5)	1.186	0.724–1.944	0.499			
**Treatment (TACE+T** *vs.* **TACE+T+I)**	1.771	1.095–2.865	0.020	1.788	1.097–2.915	**0.020**

HR, hazard ratio; CI, confidence interval; HBV, hepatitis B virus; AFP, alpha-fetoprotein; PIVKA, Protein Induced by Vitamin K Absence or Antagonist II; ALBI, albumin-bilirubin; ECOG PS, Eastern Cooperative Oncology Group Performance Status; TACE, transarterial chemoembolization; TACE+T+I, TACE + targeted agents + immune checkpoint inhibitors; TACE+T, TACE + targeted agents. P-Values less than 0.05 in multivariate analysis are shown in bold.

### 3.6 AEs

Regardless of the inducement of TACE or systemic agents, no grade 4 AEs or treatment-related death was observed during follow-up in the two groups. There were 92.9% (39/42) patients in the TACE+T+I group and 95.6% (43/45) patients in the TACE+T group who experienced TACE-related AEs, including fever, pain, transient hepatic dysfunction, and embolization-induced nausea and vomiting. All the TACE-related AEs were in grades 1–2 and showed no significant difference between the two groups. AEs related to targeted and immune agent administration are summarized in **Table 5**. Among them, the most common AEs in the TACE+T+I group were proteinuria (23.8%) and skin rash (23.8%), and in the TACE+T group, they were high blood pressure (24.4%) and skin rash (16.0%). Grade 3 AEs occurred in 8 (19.0%) patients of the TACE+T+I group compared with 7 (15.6%) patients in the TACE+T group, indicating no significant difference (*p* = 0.667). In addition, there were 9 (21.4%) patients who experienced dosage adjustment or medicine discontinuation as a result of AEs in the TACE+T+I group and 7 (15.6%) patients in the TACE+T group.

**Table 5:** Adverse events attributed to systemic agents.

Adverse events	TACE+T+I (n = 42)		TACE+T (n = 45)	
	All grade	3/4 grade	All grade	3/4 grade
RCCEP	8		7	
Skin rash	10	1	8	2
Hand-foot syndrome	5	1	6	1
Proteinuria	10	2	5	1
Hypertension	9		11	
Hypothyroidism	3		4	
Diarrhea	4		7	1
Pruritus	8		4	
Liver dysfunction	7	2	6	2
Fatigue	1		5	
Anemia	1		0	
Alopecia	0		2	
Immune hypophysitis	1	1	0	
Pneumonia	1	1	0	
	68	8 (19.0%)	65	7 (15.6%)

RCCEP, reactive cutaneous capillary endothelial proliferation.

## 4 Discussion

Our cohort research retrospectively evaluated TACE combined with molecularly targeted agents plus ICIs and TACE combined with molecularly targeted agents for intermediate and advanced HCC. The results showed that the median OS (24.00 *vs.* 21.40 months, *p* = 0.007) and the median PFS (9.70 *vs.* 7.00 months, *p* = 0.017) were both significantly prolonged in the TACE+T+I group than in the TACE+T group. Before this study, the administration of TACE plus anti-angiogenic agents has been already applied widely in real-world implementations (11–13). The TACTICS trial in Japan and the LAUNCH trial in China reported the superiority of such combination therapy (14, 15). The positive results of these two large trials may concurrently reveal the particularity of the population in Asia, in view of the etiology of most HCC patients here with hepatitis B. Moreover, seeing that HCC is considered an immunogenic tumor arising in a chronically inflamed liver, immune treatment may represent an irreplaceable therapeutic tool to achieve satisfactory effects in HCC patients (16).

Some published studies supported the theory that anti-angiogenic agents may cause vascular normalization and positive immune regulation, which could amplify the efficacy of immunotherapy (4, 17). Ji et al. conducted a retrospective comprehensive evaluation of the combination therapy of TACE, sorafenib, and ICIs. The analysis reported that compared with those in the TACE+Sor group, PFS and OS were significantly prolonged in the TACE+Sor+ICI group (median PFS, 16.26 *vs.* 7.30 months, *p* < 0.001; median OS, 23.3 *vs.* 13.8 months, *p* = 0.012) (18). The median OS observed in the triple combination group in our investigation was very similar to that of the study mentioned above, but the median PFS in the triple group of our study was obviously shorter. The possible reason might be that the inclusion criteria in Ji’s study only included TACE-refractory HCC, and the last TACE before refractory was defined as the first TACE in follow-up. Another cohort study, which only included BCLC C stage HCC, also indicated that TACE combined with lenvatinib plus PD-1 inhibitor (T+L+P) could achieve better efficacy than TACE combined with lenvatinib (T+L) with manageable toxicity (19). This result was similar to the subgroup analysis of BCLC stage C HCC patients in our study. However, while TACE+T+I also significantly prolonged the mOS of patients with BCLC stage B HCC, the mPFS showed no statistical difference in the two groups according to our results. The reason for the opposite result could be that TACE only controlled the intrahepatic lesions, while the alleviation of vascular tumor thrombus and extrahepatic metastasis mainly depends on the systemic treatment. However, our results indicated that triple combination therapy could benefit more for HCC patients at BCLC stage C rather than stage B.

The remarkable strength that emerged from TACE+T+I could be explained by the effects of different therapies in the immune microenvironment. Some studies have analyzed the changes in the HCC tumor microenvironment after TACE and found that TACE is an inducer of immunogenic cell death (20). TACE can cause tumor cell necrosis and lead to the release of neoantigens, promote the recruitment and activation of dendritic cells into the microenvironment, and transform the immunosuppressive microenvironment into an immunosupportive environment (21). In this regard, once combined with immune therapy, TACE enables immune agents to display function better in the “activated” microenvironment to achieve better efficacy. A propensity score-matched study found that compared with monotherapy (ICIs) group, patients undergoing ICIs with TACE achieved a significantly longer median PFS [8.8 months (95% CI: 6.2–23.2) *vs.* 3.7 months (95% CI: 2.7–5.4), log-rank 0.15, *p* < 0.01] and numerically longer median OS [35.1 months (95% CI: 16.1–not evaluable) *vs.* 16.6 months (95% CI: 15.7–32.6), log-rank 0.41, *p* = 0.12] (22). Some other lines of evidence suggest that VEGF not only is a pro-angiogenic factor but also plays an important role in the formation of immunosuppressive tumor microenvironment. Voron et al. found that targeted drugs could reduce the expression of VEGF-induced inhibitory receptors mediating CD8+ T-cell exhaustion (23). Other studies showed that VEGF/VEGFR signaling pathway could inhibit the function and differentiation of dendritic cells (DCs) and induce myeloid-derived suppressor cells (MDSCs) (24). In addition, VEGF can directly cause the exhaustion of TOX-dependent T cells. A mouse study showed that anti-PD-1 combined with VEGFR treatment inhibited tumor growth and survival through vascular normalization and enhanced antitumor immune responses (25). Given these results, the combination of TACE with anti-angiogenic therapy plus ICIs appears to overcome the innate tumor resistance to immune response and achieve a better therapeutic effect.

In this study, the different treatment method was the only independent factor affecting OS, and receiving the treatment of TACE+T+I was a strongly protective factor of OS, which could reduce the risk of death by approximately 60%. Moreover, BCLC stage and vascular invasion were also strong factors affecting OS in univariate analysis but changed in multivariate analysis. This resulted from the interactional effect among vascular invasion, BCLC stage, tumor size, and treatment methods. Unexpectedly, except for the independent effect of the treatment method, age < 65 was an independent risk factor of PFS. This surprising result might be due to the limited population.

As seen in **Table 2**, the ORR rate was significantly improved in TACE+T+I at 52.4% *vs.* 17.8% in TACE+T with four patients (9.5%) achieving CR and a DCR of 83.3%. A systemic review analyzed 741 patients in 15 studies who received the triple combination of TACE/HAIC, TKIs, and ICIs, and results showed that the triple combination provided a substantial CR rate of 12.4% (6.9%–19.0%), ORR of 60.6 (52.8%–68.2%), and DCR of 88.5% (83.5%–92.7%), which showed good agreement with our data. ^11^ Nevertheless, the DCR rates between the two groups in our study were nearly the same (83.3% *vs.* 77.8%, *p* = 0.514), which may indicate that the double treatment of TACE plus molecularly targeted agents has an effect on stabilizing the tumor, and the addition of ICIs achieves tumor regression on the basis of that.

The systemic agent-attributed AEs observed in this study were relatively common in previous studies (26, 27), and no grade 4 AEs or treatment-related death occurred in the two groups. The most common AEs that occurred in this study included hypertension, skin rash, proteinuria, and reactive cutaneous capillary endothelial proliferation (RCCEP), which were also similar to those in previous research. Although almost all patients in our study experienced TACE-related AEs, there were no TACE-related grade 3/4 AEs found in the two groups. A network meta-analysis analyzed various phase III trials about novel combination strategies for unresectable hepatocellular carcinoma (28). As reported in the study, grade 3 AE incidence ranged from 22.3% to 75%, and the incidence of AEs requiring treatment discontinuation ranged from 1% to 27.3%, which was comparable to ours.

Therefore, as the results indicated in our study, the triple combination of TACE, molecularly targeted agents, and ICIs can improve the prognosis of intermediate and advanced HCC better than the double combination of TACE and targeted therapy. However, some issues also need to be solved: 1) the interval between the initiation of targeted and immune therapy, 2) whether the targeted and immune therapy should be pre-TACE or post-TACE and their interval, and 3) what are the termination criteria of the combination therapy.

The study has several limitations. First, this study was a retrospective study with limited follow-up time, which inevitably causes relatively limited evidence. Second, due to a small sample and selective bias, the condition of receiving previous TACE in the baseline characteristics showed a statistical difference between the two groups, although previous TACE was not a predicted factor of OS or PFS. Third, the anti-angiogenic agents and immune agents included in our study were diverse, so it was hard to avoid the confounding effects resulting from different drug administrations.

## 5 Conclusion

The triple treatment of TACE combined with molecularly targeted agents and ICIs seems to improve the outcomes in intermediate and advanced HCC and simultaneously with manageable security. Prospective randomized controlled studies with larger samples should be conducted in the future to further explore the mechanism of combination therapy and provide reasonable guidance for HCC treatment strategies.

## Data availability statement

The raw data supporting the conclusions of this article will be made available by the authors, without undue reservation.

## Author contributions

All authors contributed to the study’s conception and design. Material preparation, data collection, and analysis were performed by NJ and JH. The first draft of the manuscript was written by NJ and BZ, and all authors commented on previous versions of the manuscript. All authors contributed to the article and approved the submitted version.

## Glossary

**Table:**

TACE	transarterial chemoembolization
uHCC	unresectable hepatocellular carcinoma
CR	complete response
PR	partial response
SD	stable disease
PD	progressive disease
OS	overall survival
PFS	progression-free survival
ORR	objective response rate
DCR	disease control rate
AEs	adverse events
HCC	hepatocellular carcinoma
PLC	primary liver cancer
BCLC	Barcelona Clinic Liver Cancer
HIF-1α	hypoxia-inducible factor-1α
T+A	atezolizumab plus bevacizumab
ICIs	immune checkpoint inhibitors
RCT	randomized controlled trial
CNLC	China liver cancer staging
CT	computed tomography
MR	magnetic resonance
mRECIST	modified Response Evaluation Criteria in Solid Tumors
ECOG	Eastern Cooperative Oncology Group
cTACE	conventional transarterial chemoembolization
DEB-TACE	drug-eluting bead transarterial chemoembolization
CTCAE	Common Terminology Criteria for Adverse Events
ALBI	albumin-bilirubin
AFP	alpha-fetoprotein
PIVKA-II	Protein Induced by Vitamin K Absence or Antagonist II
DC	dendritic cells
MDSCs	marrow-derived suppressor cells.

## References

[1] Sung H Ferlay J Siegel RL Laversanne M Soerjomataram I Jemal A. Global cancer statistics 2020: GLOBOCAN estimates of incidence and mortality worldwide for 36 cancers in 185 countries. CA Cancer J Clin (2021) 71:209–49. doi: 10.3322/caac.21660 33538338

[2] Park JW Chen M Colombo M Roberts LR Schwartz M Chen PJ. Global patterns of hepatocellular carcinoma management from diagnosis to death: the BRIDGE Study. LIVER Int (2015) 35:2155–66. doi: 10.1111/liv.12818 PMC4691343 25752327

[3] Reig M Forner A Rimola J Ferrer-Fabrega J Burrel M Garcia-Criado A. BCLC strategy for prognosis prediction and treatment recommendation: The 2022 update. J Hepatol (2022) 76:681–93. doi: 10.1016/j.jhep.2021.11.018 PMC8866082 34801630

[4] Petrillo M Patella F Pesapane F Suter MB Ierardi AM Angileri SA. Hypoxia and tumor angiogenesis in the era of hepatocellular carcinoma transarterial loco-regional treatments. Future Oncol (2018) 14:2957–67. doi: 10.2217/fon-2017-0739 29712486

[5] Lencioni R de Baere T Soulen MC Rilling WS Geschwind JF. Lipiodol transarterial chemoembolization for hepatocellular carcinoma: A systematic review of efficacy and safety data. HEPATOLOGY (2016) 64:106–16. doi: 10.1002/hep.28453 26765068

[6] Galle PR Finn RS Qin S Ikeda M Zhu AX Kim TY. Patient-reported outcomes with atezolizumab plus bevacizumab versus sorafenib in patients with unresectable hepatocellular carcinoma (IMbrave150): an open-label, randomised, phase 3 trial. Lancet Oncol (2021) 22:991–1001. doi: 10.1016/S1470-2045(21)00151-0 34051880

[7] Kelley RK Sangro B Harris W Ikeda M Okusaka T Kang YK. Safety, efficacy, and pharmacodynamics of tremelimumab plus durvalumab for patients with unresectable hepatocellular carcinoma: randomized expansion of a phase I/II study. J Clin Oncol (2021) 39:2991–3001. doi: 10.1200/JCO.20.03555 34292792 PMC8445563

[8] Xu F Jin T Zhu Y Dai C. Immune checkpoint therapy in liver cancer. J Exp Clin Cancer Res (2018) 37:110. doi: 10.1186/s13046-018-0777-4 29843754 PMC5975687

[9] Ren Z Xu J Bai Y Xu A Cang S Du C. Sintilimab plus a bevacizumab biosimilar (IBI305) versus sorafenib in unresectable hepatocellular carcinoma (ORIENT-32): a randomised, open-label, phase 2-3 study. Lancet Oncol (2021) 22:977–90. doi: 10.1016/S1470-2045(21)00252-7 34143971

[10] Xu J Shen J Gu S Zhang Y Wu L Wu J. Camrelizumab in combination with apatinib in patients with advanced hepatocellular carcinoma (RESCUE): A nonrandomized, open-label, phase II trial. Clin Cancer Res (2021) 27:1003–11. doi: 10.1158/1078-0432.CCR-20-2571 33087333

[11] Ke Q Xin F Fang H Zeng Y Wang L Liu J. The significance of transarterial chemo(Embolization) combined with tyrosine kinase inhibitors and immune checkpoint inhibitors for unresectable hepatocellular carcinoma in the era of systemic therapy: A systematic review. Front Immunol (2022) 13:913464. doi: 10.3389/fimmu.2022.913464 35677059 PMC9167927

[12] Zhao Y Wang WJ Guan S Li HL Xu RC Wu JB. Sorafenib combined with transarterial chemoembolization for the treatment of advanced hepatocellular carcinoma: a large-scale multicenter study of 222 patients. Ann Oncol (2013) 24:1786–92. doi: 10.1093/annonc/mdt072 23508822

[13] Cheng Z He L Guo Y Song Y Song S Zhang L. The combination therapy of transarterial chemoembolisation and sorafenib is the preferred palliative treatment for advanced hepatocellular carcinoma patients: a meta-analysis. World J Surg Oncol (2020) 18:243. doi: 10.1186/s12957-020-02017-0 32917226 PMC7488414

[14] Kudo M Ueshima K Ikeda M Torimura T Tanabe N Aikata H. Randomised, multicentre prospective trial of transarterial chemoembolisation (TACE) plus sorafenib as compared with TACE alone in patients with hepatocellular carcinoma: TACTICS trial. GUT (2020) 69:1492–501. doi: 10.1136/gutjnl-2019-318934 PMC7398460 31801872

[15] Peng Z Fan W Zhu B Wang G Sun J Xiao C. Lenvatinib combined with transarterial chemoembolization as first-line treatment for advanced hepatocellular carcinoma: A phase III, randomized clinical trial (LAUNCH). J Clin Oncol (2022) 41:117–127. doi: 10.1200/JCO.22.00392 35921605

[16] Ogunwobi OO Harricharran T Huaman J Galuza A Odumuwagun O Tan Y. Mechanisms of hepatocellular carcinoma progression. World J Gastroenterol (2019) 25:2279–93. doi: 10.3748/wjg.v25.i19.2279 PMC6529884 31148900

[17] Zhang B Tang B Gao J Li J Kong L Qin L. A hypoxia-related signature for clinically predicting diagnosis, prognosis and immune microenvironment of hepatocellular carcinoma patients. J Transl Med (2020) 18:342. doi: 10.1186/s12967-020-02492-9 32887635 PMC7487492

[18] Zheng L Fang S Wu F Chen W Chen M Weng Q. Efficacy and safety of TACE combined with sorafenib plus immune checkpoint inhibitors for the treatment of intermediate and advanced TACE-refractory hepatocellular carcinoma: A retrospective study. Front Mol Biosci (2020) 7:609322. doi: 10.3389/fmolb.2020.609322 33521054 PMC7843459

[19] Huang J Cai M Huang W Guo Y Zhou J Liang L. Transarterial chemoembolization combined with sorafenib and iodine-125 seed brachytherapy for hepatocellular carcinoma with portal vein tumor thrombus: a retrospective controlled study. Chin Med J (Engl) (2021) 135:113–5. doi: 10.1097/CM9.0000000000001537 PMC8850867 34507316

[20] Pinato DJ Murray SM Forner A Kaneko T Fessas P Toniutto P. Trans-arterial chemoembolization as a loco-regional inducer of immunogenic cell death in hepatocellular carcinoma: implications for immunotherapy. J Immunother Cancer (2021) 9:e003311. doi: 10.1136/jitc-2021-003311 34593621 PMC8487214

[21] Doemel LA Santana JG Savic LJ Gaupp F Borde T Petukhova-Greenstein A. Comparison of metabolic and immunologic responses to transarterial chemoembolization with different chemoembolic regimens in a rabbit VX2 liver tumor model. Eur Radiol (2022) 32:2437–47. doi: 10.1007/s00330-021-08337-3 PMC9359419 34718844

[22] Marinelli B Kim E D’Alessio A Cedillo M Sinha I Debnath N. Integrated use of PD-1 inhibition and transarterial chemoembolization for hepatocellular carcinoma: evaluation of safety and efficacy in a retrospective, propensity score-matched study. J Immunother Cancer (2022) 10:e004205. doi: 10.1136/jitc-2021-004205 35710293 PMC9204420

[23] Voron T Colussi O Marcheteau E Pernot S Nizard M Pointet AL. VEGF-A modulates expression of inhibitory checkpoints on CD8+ T cells in tumors. J Exp Med (2015) 212:139–48. doi: 10.1084/jem.20140559 PMC4322048 25601652

[24] Kim CG Jang M Kim Y Leem G Kim KH Lee H. VEGF-A drives TOX-dependent T cell exhaustion in anti-PD-1-resistant microsatellite stable colorectal cancers. Sci Immunol (2019) 4:eaay0555. doi: 10.1126/sciimmunol.aay0555 31704735

[25] Shigeta K Datta M Hato T Kitahara S Chen IX Matsui A. Dual programmed death receptor-1 and vascular endothelial growth factor receptor-2 blockade promotes vascular normalization and enhances antitumor immune responses in hepatocellular carcinoma. HEPATOLOGY (2020) 71:1247–61. doi: 10.1002/hep.30889 PMC7000304 31378984

[26] Chen S Wu Z Shi F Mai Q Wang L Wang F. Lenvatinib plus TACE with or without pembrolizumab for the treatment of initially unresectable hepatocellular carcinoma harbouring PD-L1 expression: a retrospective study. J Cancer Res Clin Oncol (2022) 148:2115–25. doi: 10.1007/s00432-021-03767-4 PMC9293824 34453221

[27] You R Xu Q Wang Q Zhang Q Zhou W Cao C. Efficacy and safety of camrelizumab plus transarterial chemoembolization in intermediate to advanced hepatocellular carcinoma patients: A prospective, multi-center, real-world study. Front Oncol (2022) 12:816198. doi: 10.3389/fonc.2022.816198 35982962 PMC9378838

[28] Fulgenzi C D’Alessio A Airoldi C Scotti L Demirtas CO Gennari A. Comparative efficacy of novel combination strategies for unresectable hepatocellular carcinoma: A network metanalysis of phase III trials. Eur J Cancer (2022) 174:57–67. doi: 10.1016/j.ejca.2022.06.058 35970037

